# Learning analysis of health system resilience

**DOI:** 10.1093/heapol/czae113

**Published:** 2024-11-22

**Authors:** Kyaw Myat Thu, Sarah Bernays, Seye Abimbola

**Affiliations:** Faculty of Medicine and Health, Sydney School of Public Health, The University of Sydney, A 27 Fisher Road, Sydney, NSW 2006, Australia; Faculty of Medicine and Health, Sydney School of Public Health, The University of Sydney, A 27 Fisher Road, Sydney, NSW 2006, Australia; Faculty of Medicine and Health, Sydney School of Public Health, The University of Sydney, A 27 Fisher Road, Sydney, NSW 2006, Australia

**Keywords:** learning, resilience, health system

## Abstract

The emergence of ‘resilience’ as a concept for analysing health systems—especially in low- and middle-income countries—has been trailed by debates on whether ‘resilience’ is a process or an outcome. This debate poses a methodological challenge. What ‘health system resilience’ is interpreted to mean shapes the approach taken to its analysis. To address this methodological challenge, we propose ‘learning’ as a concept versatile enough to navigate the ‘process versus outcome’ tension. Learning—defined as ‘the development of insights, knowledge, and associations between past actions, the effectiveness of those actions, and future actions’—we argue, can animate features that tend to be silenced in analyses of resilience. As with learning, the processes involved in resilience are cyclical: from absorption to adaptation, to transformation, and then to anticipation of future disruption. Learning illuminates how resilience occurs—or fails to occur—interactively and iteratively within complex systems while acknowledging the contextual, cognitive, and behavioural capabilities of individuals, teams, and organizations that contribute to a system’s emergence from or evolution given shocks/stress. Learning analysis can help to resist the pull towards framing resilience as an outcome—as resilience is commonly used to mean or suggest a state or an attribute, rather than a process that unfolds, whether the outcomes are deemed positive or not. Analysing resilience as a learning process can help health systems researchers better systematically make sense of health system responses to present and future stress/shocks. In qualitative or quantitative analyses, seeing what is to be analysed as ‘learning’ rather than the more nebulous ‘resilience’ can refocus attention on what is to be measured, explained, and how—premised on the understanding that a health system with the ability to learn is the one with the ability to be resilient, regardless of the outcome of such a process.

1

Key messages

- What ‘health system resilience’ is interpreted to mean shapes the methodological approach taken to its analysis. Current debates on whether it is a ‘process’ or an ‘outcome’ pose a methodological challenge in efforts to analyse it. We propose ‘learning’ as an analytical concept that can help to navigate the tensions between the process versus outcome framings of health system resilience.
- Methodologically, a learning analysis of resilience offers three sets of mechanisms that may be used to explain the responses (or lack thereof) of health system actors to shocks and stress: (I) single-loop learning, (II) double-loop learning, and (III) triple-loop learning. It also offers processes or means of learning (i.e. through information, deliberation, and action) that may be working within each mechanism. These three mechanisms provide starting points for inquiry, such that to explain resilience then is to understand the contextual factors that enable or constrain (the processes embedded within) each mechanism or loop of learning.
- A learning analysis of resilience signals an important methodological shift in that it recognizes a spectrum of potential states that may be described as resilience (e.g. whether the system is functioning at 10%, 20%, or 65%) and focuses attention instead on explaining the outcomes of resilience processes, not as optimal end points—i.e. the outcome does not have to be 100% or 120% to be deemed resilience. Thus, resilience becomes a process to be understood and a state to be explained in efforts to improve how health systems function—not an outcome or destination.
- Learning analysis of resilience opens the concept up, methodologically, to careful analysis: in the kinds of questions we pose to a system; whose ‘resilience’ we seek to explain; the kinds of processes we seek to unravel and at which level (individual, team, organizational); the mechanisms through which resilience may happen; the contextual factors that may enable or constrain each mechanism (and processes embedded within each mechanism); and in the openness it allows to various potential outcomes of such mechanisms, whether positive or negative, desired or not.

## Introduction

Resilience has become a buzzword, first after the 2013–14 Ebola outbreak in West Africa and even more so in the wake of the Coronavirus disease pandemic. Various researchers and disciplines frame the concept differently, and health system actors implement strategies to optimize resilience in different ways, with varying outcomes (Jung et al. 2021, Bollyky et al. 2022, Neill et al. 2023). Semantics matter. The lack of a conceptually clear and practically actionable framing of resilience limits researchers’ ability to analyse how and why those strategies work differently across settings, or to design and implement strategies, or to learn and transfer insights across settings. It poses a methodological challenge in health systems research. Definitions and framings shape the way that we imagine, measure, and explain what might be required for resilience and how resilience could be achieved in different settings. What we interpret ‘health system resilience’ to mean will inevitably shape the approach taken to its analysis. To leave that challenge unresolved is to leave a methodological question unanswered.

We therefore offer our methodological musings on how ‘learning’—i.e. ‘the development of insights, knowledge, and associations between past actions, the effectiveness of those actions, and future actions’ (Fiol and Lyles 1985, Sheikh and Abimbola 2021)—can help health systems researchers navigate the tensions, especially between the framing of health system resilience as a process versus an outcome, enhance our understanding of what health system resilience means, and illuminate ongoing debates on how it should be analysed, given that learning is central to how health system actors (as individuals or groups, teams, and organizations) respond to shocks/stress.

## The tension between resilience as a process versus an outcome

There is a growing body of health systems literature offering various critiques of the framing of resilience, with methodological consequences. These critiques—and suggestions for how health system resilience should be framed and analysed—are made largely through a lens that refracts resilience into either a process or an outcome. However, in this ‘process versus outcome’ tension, there is a gravitational pull towards a static conceptualization of resilience as an outcome. Indeed, resilience is commonly used to mean or suggest a state or an attribute, rather than a process. The debate on what resilience means or how the concept ought to be used has also involved the use of other terms and framings to characterize the tension: emergent versus static, negative versus positive resilience, or adaptation without robustness (coping) versus adaptation with robustness (resilience) (Table 1).

**Table 1.** The tension between resilience as a process versus an outcome

Outcome	Process
The framing of health system resilience as an outcome defines it in terms of ability ‘to prepare and respond to crises while maintaining basic services, adapting and learning from external shocks’ (p. 192) (Neill et al. 2023) and therefore as something about which evaluative assessments may be conducted (Kruk et al. 2015; Neill et al. 2023; Topp 2023) and premised on the notion that ‘resilience—a normative good—is the product of adaptive responses’ to shocks and stress (Topp 2023). Such evaluations can take the focus away from explaining the outcomes (Turenne et al. 2019; Topp 2020; Neill et al. 2023), drive researchers’ and policymakers’ attention to visible, easily quantifiable (hardware) elements of the health system (Topp 2023) at the expense of intangible and hard-to-quantify (software) elements, and ignore the dynamic interactions among health system actors in response to evolving challenges (Barasa et al. 2017a; Neill et al. 2023; Topp 2023). The framing of resilience as an outcome has also been described as consistent with taking the term to mean a static feature of a health system, although resilience is merely a product of absorption and adaptation in the face of shock (Topp 2023), which undermines focus on the emergent nature of health system resilience, narrowly views systems as linear and static (Folke 2006; Barasa et al. 2017a), poorly recognizes health systems as complex adaptive systems (CASs) with nonlinear dynamics and capacity to rearrange, self-organize, or transforms as necessary to respond to external or internal shocks and stress (Martin and Sunley 2007; Bristow and Healy 2014). There is also a tendency for the framing of resilience as an outcome to suggest that it is necessarily a positive, desirable state and that resilience, by default, represents a measurable positive state of a health system and an expected and presumably a good outcome that is derived from or the result of response to shocks and stress when a system is governed well by a set of values (Topp 2023) or has a set of other necessary attributes. This positive framing, again, can undermine the understanding and analyses of health systems, as with resilience, as complex and adaptive and not static. The framing of resilience as an outcome can also suggest that resilience simply means bouncing back from shock and stress to their original state (van de Pas et al. 2017; Topp 2020, Topp 2023; Folke 2006; MacKinnon and Derickson 2013; Barasa et al. 2017a) while neglecting whether the original state in terms of quality of service, coverage, costs, etc. is desirable. Or that systems can be resilient, while certain individuals or groups (especially community and front-line health workers) stretch themselves ever so further to withstand or adapt to a situation without support to absorb, adapt, or transform the *status quo* (Gore 2019; Schneider et al. 2022; Topp 2023)	This framing of health system resilience as a process focuses attention on explanations of how and why change occurs or fails to occur (Topp 2023), including the role of power, interests, and learning within the health system—and therefore, this framing lends itself readily to system thinking (Blanchet 2015)—thus producing ‘explanatory accounts of change (or resistance to it)’ (Topp 2023) as opposed to or in tension with evaluative assessments. This framing of resilience as a health system process—which include strategies that are deployed from time to time to maintain or improve health system functions (Lerosier et al. 2023)—can help health system researchers, practitioners and policymakers explore how and why certain changes occur and inform how they might devise context-sensitive strategies to optimize health system response to shocks and stress (Peters 2014; Topp 2023). The framing of resilience as a process recognizes that resilience emerges from interactions among system actors within CASs—rather a linear cause-and-effect state (Béné et al. 2012; Kruk et al. 2015; Barasa et al. 2017a; Barasa et al. 2018)—in response to constantly shifting and changing contexts and the emergence of future states (desirable or undesirable) during and outside periods of shocks and stress. Learning is thus central to ‘the resilience dividend’ (Fleming 2015) during and after crisis (Masten 2001; Kruk et al. 2015) and day-to-day (Thomas et al. 2013; Kieny et al. 2014; Kruk et al. 2015; Barasa et al. 2017a). The framing of resilience as a process can include a continuum ranging from positive to negative, such that when negative, rather than anticipate, absorb, adapt, and transform in response to shocks (positive resilience), health systems may respond with inaction, inertia, resignation, and degradation (negative resilience) (Lerosier et al. 2023). As such, health systems can maladapt to undesirable states (Barasa et al. 2017a;Barasa et al . 2017b; Gilson et al. 2017). This understanding of resilience as a continuum is consistent with the framing of resilience as emergent. The framing of resilience as a process recognizes that resilience is not necessarily positive and draws attention to power dynamics and structural determinants of how health systems respond to shocks and stress and especially to the requirement of robustness in the resources and design of a system (Abimbola and Topp 2018), as a necessary precondition for health system resilience processes or learning processes to be positive; otherwise the system is just coping, as, typically, less powerful actors within the system overstretch themselves in response to shocks and stress to keep it functioning, even if suboptimally whether episodic or occurring day-to-day.

To navigate the tension between framing resilience as a process versus resilience as an outcome, health systems researchers may benefit from an analytical approach that is attentive to the need for systems thinking (recognizing that health systems are complex and adaptive systems), the need to attend to power (e.g. in who decides whether a system is positively resilient or in who is responsible for the contextual weaknesses that make a system negatively resilient), and the need to analyse resilience as an active rather than a passive phenomenon. We argue that ‘learning’ is an analytically and practically potent concept that can help navigate the ‘process versus outcome’ tension, providing a methodological entry point that can help to limit or avoid the risk of being drawn or pulled towards the static conceptualization of resilience and more towards the emergent nature of resilience.

## Learning as resilience

Health systems continuously experience stress and shocks. As a result, health systems everywhere have a rich—if typically undocumented—experience of going through, adapting, reorganizing, and reorienting in response to current and future challenges (Blanchet 2015). At the heart of that process is learning—i.e. learning can explain how and why changes occur at the system level and what makes changes differ over time or from one setting to another (Blanchet 2015, Topp 2023). Learning provides a useful analytical lens to examine past changes, current actions, and anticipatory efforts in different contexts and to analyse the dynamic interactions among health system actors at individual, team, or organizational levels that influence responses to shocks and stress (Blanchet 2015).

To respond is to learn or to miss an opportunity to learn. Learning is underpinned by active interaction with challenges, context, and capabilities of actors (Lengnick-Hall and Beck 2005, Barasa et al. 2017a, Abimbola et al. 2019, Abimbola 2022). Learning illuminates how resilience occurs interactively and iteratively, as the behaviour of individuals, teams, and organizations contribute to evolution or emergence from threats (Fiol and Lyles 1985, Barasa et al. 2017a, Abimbola 2022). Learning draws attention to health system capabilities for awareness, which are necessary to adjust its regular activities, practices, or structures to survive episodic challenges or the continuous improvement necessary to maintain day-to-day performance.

Learning reflects power dynamics. On the one hand, change occurring through learning, especially transformational change, realigns power dynamics among individuals, groups, and organizations. On the other hand, learning can also embed and consolidate power in the same way that resilience can manifest to protect a normatively bad outcome (Walker et al. 2004, Blanchet 2015). Learning also highlights the ability to apprehend different forms of knowledge and experience—knowledge of previous responses is instrumental in responding to current threats or preparing for future shocks. Using the concept of learning to analyse health system resilience can show how different actors use and make sense of knowledge and other resources to respond to current and future shocks. Health systems with the capacity to learn are health systems with the ability to optimize resilience.

## How learning and resilience processes align

**Figure 1.**
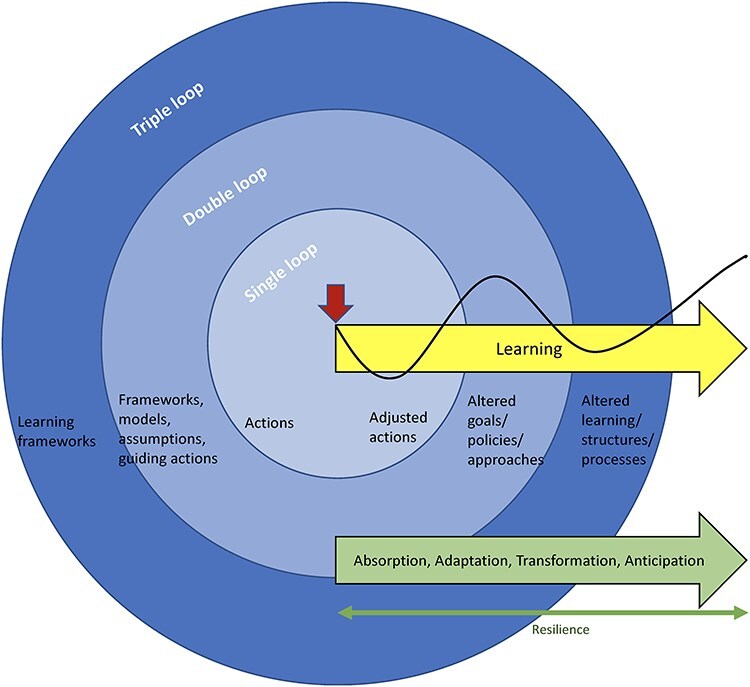
An illustration of how learning is closely linked to the resilience of a health system.

**Figure 2.**
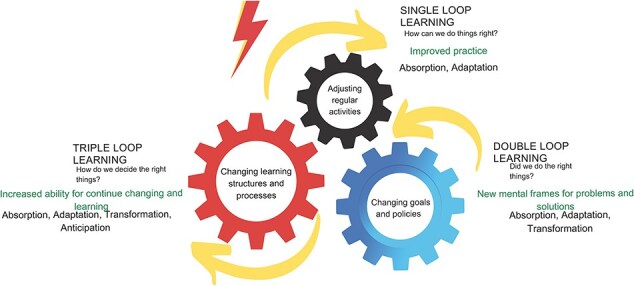
An illustration of the three loops of learning and their link to resilience processes.

As with learning, the process of resilience is cyclical or iterative: from absorption to adaptation, then to transformation, and then anticipation of future disruption (Fig. 1). A learning analysis of resilience offers three sets of mechanisms that may be used to explain health system responses (or lack thereof) to shocks and stress: single-loop learning, double-loop learning, and triple-loop learning. These three mechanisms provide starting points for inquiry, such that to explain resilience is to understand the contextual factors that enable or constrain each mechanism or loop of learning. Health system capacities that are currently used to analyse resilience—i.e. capacity for absorption, adaptation, transformation, and anticipation—represent facets of resilience or how resilience process may unfold over time and through events, but are not generative mechanisms that may explain it. As shown in Figs 1 and 2, single-loop learning, double-loop learning, and triple-loop learning do not map neatly onto absorption, adaptation, transformation, and anticipation capacities.

The downward arrow at the centre of the loops represents a health system shock or stress. The waving line illustrates the process of ‘resilience’ by which a health system might respond to a shock or stress: absorption, adaptation, transformation, and anticipation of future disruption. The arrow, which signifies learning, explains the processes involved in resilience (depicted by other two arrows) as cyclical and iterative. These three loops of learning—single-loop, double-loop, and triple-loop learning—serve as mechanisms to explain facets of health systems’ responses/resilience to shocks and stresses.

The lightning bolt represents a shock or stress experienced by a health system. The curved arrows illustrate that cyclical and iterative process of learning, represented by three loops of learning in the shape of wheels. These three loops embody the mechanisms involved in the learning that may generate the processes that characterize health system resilience—i.e. absorption, adaptation, transformation, and anticipation of future disruption.

Single-loop learning typically occurs at the individual level, as front-line health workers, managers, community members, or any individual in the team or organization make adjustments to their regular actions (in response to errors, failures, challenges, shocks, stress, etc.) without checking assumptions or root causes. For example, a village leader may repair a bridge that was destroyed by a flood to maintain access to the health facility and thus to maintain existing levels of childhood immunization in the village, without questioning the *status quo* of low childhood immunization coverage in the community. Single-loop learning manifests in the form of absorptive capacity as actors change their daily routines and find *ad hoc* solutions to sustain performance or survive current challenges (Fig. 1). Like absorptive strategies, single-loop learning tends to only enable health systems to continue functioning at the same level with the same resources and capacity. Single-loop learning can also play a role in the adaptation and anticipation processes that may contribute to health system resilience (Fig. 1).

Double-loop learning occurs when individuals, teams/groups, or organizations examine root causes, question their assumptions, or change their approach to problems and their solutions, sometimes as a result of suboptimal outcomes of single-loop learning. For example, the village leader and the health facility manager in the flood-affected village may come together and discuss how to do things more sustainably and effectively and especially to alter the *status quo* of low childhood immunization coverage in the village. Collective actions from double-loop learning can improve system adaptation and innovation but require system actors’ capacity to be more critical of existing rationale, objectives, or assumptions behind their current practice or pvcrevious single-loop learning (reorganization of the team, reorientation of resources, obtaining complementary resources, change of policy or goal, etc.). This reflects adaptive capacity (Fig. 1). Like single-loop learning, effects of double-loop learning may stop at adaptability, as health systems rely on stretching existing limited resources to deliver the same level of services to deal with a disruption (Blanchet 2015, Lerosier et al. 2023)—i.e. coping.

The response may, however, be transformative when double-loop learning occurs in a robust context in which resources are available to reorganize services, with the capacity to reimagine rationale and assumptions. In a hypothetical example, a learning platform may be created to coordinate efforts and initiatives among health system actors, to gain better information and insight, and to learn from the village and other villages’ efforts on childhood immunization, with resulting ongoing and iterative successful efforts to achieve national and regional targets in childhood immunization coverage. Such a system can perform at a higher level than before a shock or stress event and thus show transformative capacity. But to deal with day-to-day challenges or shocks, health systems must also increase their ability to continue changing and learning. Triple-loop learning, which occurs with questioning existing learning frameworks, structures, and processes, is also essential for transformation capacity (Fig. 1). Triple-loop learning—that is, learning how to learn—puts the system in a position to anticipate future stresses or shocks—i.e. to predict and prepare for future disruption, with absorptive and adaptive response to those events if and when such shock and stress events occur (Fig. 1).

## Illustrating learning as resilience

To illustrate how a learning analysis of resilience may be conducted using these three mechanisms as the starting point of analysis, we constructed a scenario. This scenario is stylised, and it reflects an unfolding of events along the positive spectrum of resilience. But the events could easily unfold in multiple different positive or negative ways. We encourage the reader to imagine such alternative unfolding of events, and the contextual factors that may push them in one direction or the other.

The scenario is as follows: an organization (say, a 10-year-old District Health Department) has been implementing the immunization programme for children under 5 years in Village A since its creation. Immunization coverage for children under-5 years in Village A has been 10% each year since the organization started delivering the services. Political, geographical, and financial difficulties, community awareness, etc. constrained the achievement of higher immunization coverage. Recently, the immunization programme faced a significant interruption in service delivery due to a flood, which led to a breakdown of the wooden bridge that connects the city and the village.

After the acute event of the flood and bridge collapse, parents, the health facility manager, and the village leader were worried about interruption of immunization for children in the village. In the past, they had experienced a measles outbreak 6 months after discontinuing routine immunizations in the rainy seasons. The lesson learnt from that experience made the community and the manager anticipate interruption of health services in the rainy seasons and prepare to maintain access to health services (anticipation). The overall objective of the parents, community leader, and manager is restoring the normal function of immunization services, to keep the 10% coverage for under-5 children in Village A using existing resources and available capacity (absorptive strategies). Although they had similar objectives, each actor used different absorptive strategies: the village leader sought to repair the bridge in a short time; the manager’s idea was to use a boat for alternative vaccine transportation, and a minority of parents with the resources and capacity to afford it travelled to the city for immunization.

This ability of agents or actors to change their daily routines and find *ad hoc* solutions to survive the challenges allowed the continuation of services and maintained the 10% coverage of the programme in the village. But while their actions provided the desired results, they had no effect on increasing the coverage of unimmunized children in the community. This absorptive change is an example of single-loop learning, which ‘can support changes in regular actions by adapting normal routines and practises, but tends to overlook the assumptions on which these are based’ (Sheikh and Abimbola 2021). Single-loop learning in health systems involves front-line health workers, managers, the community, or any individual in the team or organization making adjustments in regular actions without checking assumptions or root causes. Absorptive changes from single-loop learning can mask root causes or systemic challenges, such as, low immunization coverage, community awareness, and accessibility issues in this scenario.

Now let us imagine that after reducing the consequences of service interruption, the community, village leader, and manager started to question whether they had taken the right actions in response to the situation. Majority of parents, who had not fully appreciated the importance of immunizing their children, whose children had not received any immunization before, observed that a small group of parents—those who recognised the value of immunization and had the resources to provide the immunization to their children— made a concerted effort to ensure their children were vaccinated, even during the breakdown of the wooden bridge. Parents in the majority group began to demand that the District Health Department extend services to their parts of the village. In an example of double-loop learning, the manager and village leader realized that 10% of immunization coverage in their village is not a worthy goal, and the responses were not sustainable for future events.

The community (now including parents with limited prior engagement with immunization services), the village leader, and the manager from Village A came together and discussed how to do things more sustainably and effectively (adaptive strategies). The experience with changes at the team/group level opened everyone’s eyes to the importance of participation and awareness in the community. The village leader and parents took action to ensure that all villagers are involved or aware of the immunization services, and the village leader and manager worked together to increase the scope of immunization services in the village (innovation). Adaptive capacity led to coordinated strategies, e.g. reorganization of service delivery, reorganization of community groups, and reorientation of resources. These efforts contributed to achieving 20% immunization coverage in the following reporting period. They could not achieve >20% coverage because the manager was not able to secure additional supplies of vaccines from the government. But the village leader working with high-income community members raised funds and built a concrete bridge (anticipatory change arising from double-loop learning).

Achieving 20% immunization coverage along with a new concrete bridge and improving community awareness and engagement resulted from double-loop learning. But the community wanted more. Through the village leader, they decided to advocate to policy makers at the district level to organize regular coordination meetings between village representatives and policy makers as a learning platform for both sets of actors. The decision on the meetings was made upon procedural changes at the organizational level, i.e. the District Health Department. They also set up a village health committee (with broad representation across different groups in the village) to meet regularly to deliberate on how to continuously improve health in Village A. The two learning platforms reflect triple-loop learning. As a result of deliberations on these two platforms, the manager submits a proposal to the District Health Department for additional resources to expand immunization and meet unmet demand and other health services in Village A (transformative strategies).

The two learning platforms allowed for the incorporation and deliberation of information from different sources, advocacy, and questioning existing practices. These practices, knowledge, and experiences were shared with other villages in the district and with other districts in the province. Service availability, accessibility, and community engagement improved dramatically after opening a new primary healthcare facility alongside ongoing regular coordination among stakeholders. All these actions contributed to achieving national and regional targets in coverage of under-5 vaccination. By the following year, the coverage of immunization services among children under 5 years in the village had gone up to 65%. Triple-loop learning supports the ability of the health system to sustainably transform its functions and structures in response to a disruption—i.e. transformative capacity.

The three means of learning—information, deliberation, and action—interplayed throughout the response. Adjustments of regular actions that occur in silos or at the individual level may be based on information or resources available at the individual level to restore or maintain normal functions without checking assumptions or root causes (Fig. 2). The decisions/actions to repair the wooden bridge, use a boat to transport vaccines, and access immunization in the city were taken based on individual intuition, interpretation, or use of available information/resources just to ensure that the children have access to vaccination without expecting the longer term or sustainability of the action. Working as a team or group (deliberation) helped actors to learn from each other, use previous experience (information), and take collective actions to change strategy (e.g. to build a concrete bridge). This double-loop learning supported system’s adaptivity and innovation (Fig. 2). Deliberation and repeated practice (action) offers the opportunity to collectively question, refine, and change existing approaches to learning, thus improving performance, adaptability, innovation, anticipation, and self-reliance (triple-loop learning) (Fig. 2). Regular coordination meetings were initiated when actors at all levels learnt the benefits of the services and used meetings to transform the system with stakeholder awareness, participation, and collaboration for continuous change and learning (information, deliberation, and action).

Whether assessed quantitatively or qualitatively, a learning analysis of resilience signals a potentially important new methodological entry point for health system researchers and other scholars of resilience. It goes beyond the tension between the framing of resilience as a process versus the framing of resilience as an outcome. It recognizes a spectrum of potential states that may be described as resilient (whether the system is functioning at 10%, 20%, or 65%)—which is consistent with the framing of resilience as an outcome. But instead of focusing on these outcomes or interpreting them as quantitative indicators of resilience, a learning analysis seeks to explain and understand how outcomes came to be and why. A learning analysis embraces the importance of outcome measures but primarily seeks to understand the processes that generated those outcomes. In doing so, a learning analysis focuses attention on understanding the outcome of resilience processes, not as optimal end points—i.e. the outcome does not have to be 100% or 120% to be deemed resilience. Such a contention is analytically less relevant. A learning analysis of resilience relieves resilience of the burden to represent an optimal state of affairs, but rather as a learning process. That a system has learnt does not necessarily mean that it functions optimally, nor should resilience. It turns resilience into a process to be understood and a state to be explained—in efforts to improve how health systems function.

Another methodological contribution of a learning analysis to health system resilience is that it offers, at a more granular level, what may be working within each mechanism or loop of learning—such as the means of learning: through information, deliberation, and action. A learning analysis opens the concept up, methodologically, to careful analysis: whether in the kinds of questions we pose to a system whose ‘resilience’ we seek to understand or explain, the kinds of processes we seek to unravel and at which level (individual, team, and organizational), the mechanisms through which resilience may happen (three loops of learning), the contextual factors that may enable or constrain each mechanism (and processes embedded in each mechanism), or the openness to various potential outcomes of such mechanisms (i.e. various potential outcomes of resilience), positive or negative, desired or not.

## Conclusion

Learning—as a concept—makes resilience methodologically legible by providing an entry point to the analysis of health system resilience that focuses on explaining processes. Framing resilience as learning can help to better analyse it as a process. Health systems can adapt to a better state or maladapt to an even worse state. Analysing resilience as learning can illuminate the complex and never-ending process of adaptation and interaction among health system actors and explain why a health system generates specific responses or outcomes routinely or in response to stress/shocks and what influences their response at different levels. Learning analysis offers the opportunity to understand how health system actors at individual, team, and organization levels respond to stress/shocks. A health system with the ability to learn is the one with the ability to be resilient, regardless of the outcome of such a process.

## Data Availability

No new data were generated or analysed as part of this work.

## Reflexivity statement

The authors are from different regions of the world — two from Low- and Middle-Income Countries and one from High-Income Countries — and have diverse experiences in health systems and global health research, which are reflected in the perspectives shared in this article.
